# Prognostic value of diabetes mellitus for acute type B intramural hematoma patients undergoing endovascular repair

**DOI:** 10.3389/fcvm.2025.1683361

**Published:** 2025-11-27

**Authors:** Fumin Yu, Miao Miao, Bin Wang, Haiying Xian

**Affiliations:** 1 Department of Cardiovascular Surgery, Guangdong Provincial People's Hospital, Guangdong Academy of Medical Sciences, Southern Medical University, Guangzhou, Guangdong, China; 2 Department of Endocrinology, Harrison International Peace Hospital, Hengshui, Hebei, China; 3 Department of Cardiovascular Surgery, Harrison International Peace Hospital, Hengshui, Hebei, China

**Keywords:** intramural hematoma, Stanford type B, Diabetes Mellitus, TEVAR, Aortic remodeling, Major adverse events

## Abstract

**Objective:**

Thoracic endovascular aortic repair (TEVAR) is increasingly performed in patients with acute type B intramural hematoma (IMH). This study aimed to evaluate the effect of diabetes mellitus (DM) on postoperative clinical outcomes and aortic remodeling degree of acute type B IMH patients undergoing endovascular repair.

**Methods:**

We retrospectively identified patients diagnosed with acute type B IMH at two medical centers between January 2017 and January 2024. Study subjects were divided into DM and none DM (nDM) groups. Baseline characteristics, procedure details, and postoperative outcomes were extracted for further analysis.

**Results:**

Forty-four patients were included in the study and were divided into DM (*n* = 12) and nDM groups (*n* = 32). Preoperative demographic characteristics, laboratory biomarkers, and imaging data were similar between the two groups. Compared with nDM group, the DM group presented a slightly higher rate of patients without postoperative major adverse events (MAE) (75.0% vs 68.8%, *p* = 0.651). At the 1-year follow-up, the DM group exhibited a significantly higher degree of aortic remodeling, as assessed by the TAD/TLD ratio [total aortic diameter [TAD] divided by the true lumen diameter [TLD] at the maximal IMH thickness] (DM group: 1.09 ± 0.04; nDM group: 1.17 ± 0.10, *p* = 0.032). Cox multivariate regression revealed that a TAD/TLD ratio > 1.32 increased the incidence of postoperative MAE significantly.

**Conclusions:**

DM is positively associated with the prognosis of patients with acute type B IMH undergoing TEVAR and promotes postoperative aortic remodeling. Moreover, a TAD/TLD ratio >1.32 independently predicts the incidence of postoperative MAE.

## Introduction

Aortic intramural hematoma (IMH) is defined as the presence of blood within the aortic wall media, potentially resulting from the rupture of vasa vasorum within the aortic wall (1, 2). For acute Stanford type B IMH not involving the ascending aorta (1, 2), optimal medical treatment (OMT) was traditionally considered the best choice (3). However, with the high failure rates of OMT (4), thoracic endovascular repair (TEVAR) has been introduced and increasingly performed in patients with suitable indication, achieving better results (1, 2, 5, 6). Nevertheless, TEVAR outcomes are not always satisfactory and can be influenced by several factors such as the timing of endovascular repair and diabetes mellitus (DM) (7–12).

DM is a common characteristic among patients with IMH undergoing the TEVAR procedure, but its impact on prognostic outcomes remains controversial. Some studies have reported that DM may reduce the incidence of major adverse events (MAE) following the TEVAR procedure (9, 10), whereas other studies have shown conflicting results (11, 12).

In some previous studies, the degree of postoperative aortic remodeling, as assessed by imaging data, was positively associated with the prognosis following the TEVAR procedure (7, 13–15). In patients with aortic aneurysms, DM may reduce the incidence of MAE following the TEVAR procedure by protecting the aortic wall and prohibiting aortic enlargement (10, 16–18). However, it remains unclear how DM affects postoperative aortic remodeling of patients with IMH undergoing TEVAR. Thus, we conducted this study to evaluate the effect of DM on patients with acute type B IMH undergoing the TEVAR procedure and to assess the potential association of DM with postoperative aortic remodeling.

## Methods

This study was authorized by the Guangdong Provincial People's Hospital's and Harrison International Peace Hospital's Ethical Committees. Informed consent was not required given the retrospective nature of this study. We utilized existing patient data, which posed minimal risk to the patients.

### Study patients

We retrospectively evaluated individuals diagnosed with IMH at Harrison International Peace Hospital and Guangdong Provincial People's Hospital between January 2017 and January 2024. Finally, patients with acute Stanford type B IMH who had undergone the TEVAR procedure were included for further analysis. The exclusion criteria were as follows: (1) Stanford type A IMH; (2) chronic IMH; (3) trauma-induced entity; and (4) treatment other than TEVAR. Then, based on the presence of DM before operation, we divided the patients into DM and none DM (nDM) groups. In accordance with guidelines, all patients received standard management upon admission, including sedative therapy, pain relief, heart rate control, and antihypertensive medication (1–3). During hospitalization, all the patients underwent postoperative aortic computed tomography (CTA) once or twice.

### TEVAR procedure

To undergo the TEVAR procedure, the patients must meet one or more of the following indications: (1) maximum diameter of the aorta within the IMH ≥ 50 mm; (2) maximum IMH thickness ≥11 mm (1, 2). The assessment for the TEVAR procedure was performed by a multidisciplinary team consisting of cardiac surgeons, anesthesiologists, and radiologists. All patients underwent the TEVAR procedure in a hybrid operating room under general anesthesia. The criteria for procedural success were as follows: (1) successful delivery to the target lesion; (2) accurate deployment of the endograft; (3) absence of type I or III endoleaks, graft obstruction, and death during procedure (1, 2). The proximal landing zone of the aortic stent graft should extend at least 20 mm beyond the aortic segment unaffected by the IMH, combined with no more than 10% stent oversizing (1, 7).

### Definitions

DM was diagnosed based on fasting plasma glucose levels ≥126 mg/dL, casual plasma glucose levels ≥200 mg/dL, hemoglobin A1c levels ≥6.5%, or treatment with anti-diabetic therapy (19, 20). We defined hypertension as systolic blood pressure (SBP) ≥ 140 mmHg, diastolic blood pressure (DBP) ≥ 90 mmHg, or treatment with antihypertensive therapy (21). Dyslipidemia was defined as follows: (1) low-density lipoprotein (LDL) cholesterol levels≥140 mg/dL; (2) high-density lipoprotein (HDL) cholesterol levels <40 mg/dL; (3) non-HDL cholesterol levels ≥170 mg/dL; (4) triglyceride levels ≥150 mg/dL; (5) or treated with antidyslipidemia therapy (22).

Based on the classification of IMH period (acute and subacute), the timing of TEVAR was determined using a cutoff of 7 days from symptom onset to intervention. Acute TEVAR (aTEVAR) was defined as intervention occurring within 1–7 days, while delayed TEVAR (dTEVAR) was defined as intervention occurring between 8 and 90 days (23).

**Figure 1:**
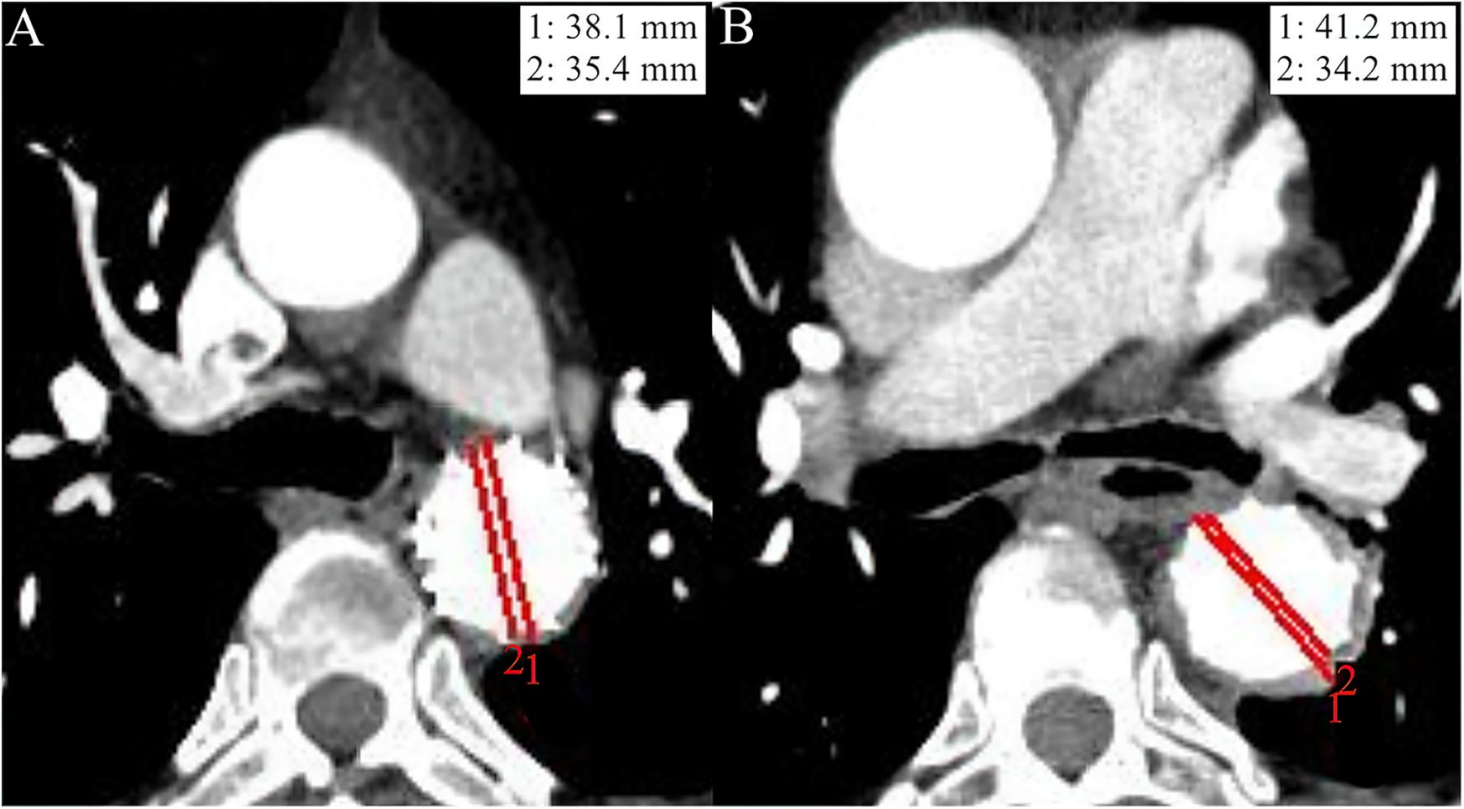
Imaging data from the aortic computed tomography angiography (CTA) of two study patients at the 1-year follow-up. The red line 1 indicates the total aortic diameter (TAD), while the red line 2 indicates the true lumen diameter (TLD). **(A)** An adult patient diagnosed with diabetes mellitus (DM) at admission had a TAD/TLD ratio of 1.08, indicating a high degree of aortic remodeling. **(B)** An adult patient without DM at admission showed relatively poor aortic remodeling degree as evaluated by a TAD/TLD ratio = 1.21, compared with patient A.

On aortic CTA, the degree of aortic remodeling was evaluated using the ratio of the total aortic diameter (TAD) to the true lumen diameter (TLD) at the site of maximum IMH thickness (TAD/TLD ratio). TLD was defined as the distance between intimal surface, while the TAD was measured from adventitia to adventitia (Figure 1). A TAD/TLD ratio closest to 1.0 was considered indicative of better aortic remodeling (7).

As clinical endpoints, we defined MAE, which included all-cause mortality, endoleak types I-IV, aortic rupture, new-onset aortic dissection (AD), re-intervention, spinal cord ischemia (SCI), stroke, and acute cardiac insufficiency (ACI).

### Follow-up

We recommended that all patients undergo aortic CTA at 1, 3, 6, and 12 months after the intervention. Subsequently, we conducted a 1-year follow-up via telephone inquiry or outpatient service. Outcomes related to clinical endpoints and imaging data were recorded for analysis.

### Statistical analyses

Continuous variables were presented in the form of mean ± standard deviation (M ± SD). Depending on the presence or absence of normality, the Student's *t* test or Mann–Whitney *U*-test was employed to compare the statistical difference. Dichotomous variables were expressed as frequency and percentage [n (%)]. Based on the sample size, we performed the Chi-square test or the Fisher's test for statistical analysis. We used the Kaplan–Meier (K-M) method to evaluate the 1-year outcomes of patients without postoperative MAE in each group. The optimal cutoff point for continuous data was determined based on previous studies. The log-rank test was used to compare statistical difference between K-M curves. We conducted Cox multivariate regression analysis to evaluate the association between variables (with a log-rank *p* value < 0.05 or traditionally recognized as risk factors) and postoperative MAE. The outcomes were presented as hazard ratio (HR) with corresponding 95% confidence intervals (CIs). Statistical analysis was performed using SPSS 26.0 software (IBM, White Plains, NY, USA), and a *p* value < 0.05 was considered to indicate a significant difference.

## Results

### Baseline characteristics

We identified 92 patients diagnosed with IMH at Harrison International Peace Hospital and Guangdong Provincial People's Hospital between January 2017 and January 2024. Forty-eight subjects were excluded because they met the exclusion criteria. Ultimately, 44 patients with acute type B IMH who had undergone TEVAR were included in this study. Twelve patients diagnosed with DM formed the DM group, while the remaining patients were assigned to the nDM group (*n* = 32).

Among the baseline characteristics, hypertension presented as the most frequent comorbidity, with no significant difference between the DM group and nDM group (91.7% for DM vs 90.6% for nDM, *p* = 0.915) (Table 1). There were no significant differences in the demographic characteristics, laboratory biomarkers, and imaging data between the two groups before TEVAR.

**Table 1:** Baseline characteristics and operation details in the two groups.

Variables	Overall	DM *n* = 12	nDM *n* = 32	*P-value*
Age, years	62.8 ± 10.3	61.1 ± 10.2	63.5 ± 10.5	0.501
Gender, male	36 (81.8)	9 (75.0)	27 (84.4)	0.473
Hypertension	40 (90.9)	11 (91.7)	29 (90.6)	0.915
SBP on admission, mmHg	147.8 ± 27.9	146.0 ± 25.7	148.4 ± 29.1	0.800
DBP on admission, mmHg	86.9 ± 17.9	87.3 ± 14.9	86.8 ± 19.1	0.925
Antidiabeitc drugs	12 (100.0)	12 (100.0)	0 (0.0)	< 0.001
Insulin	2 (16.7)	2 (16.7)	0 (0.0)	0.070
Oral medication	12 (100.0)	12 (100.0)	0 (0.0)	< 0.001
Dyslipidemia	4 (9.1)	2 (16.7)	2 (6.3)	0.284
Smoking	24 (54.5)	7 (58.3)	17 (53.1)	0.757
COPD	1 (2.3)	0 (0.0)	1 (3.1)	1.000
Chronic kidney disease	1 (2.3)	1 (8.3)	0 (0.0)	0.273
Coronary artery disease	11 (25.0)	4 (33.3)	7 (21.9)	0.434
Penetrating aortic ulcer	10 (22.7)	1 (8.3)	9 (28.1)	0.163
Prior aortic surgery	2 (4.5)	0 (0.0)	2 (6.3)	1.000
White blood cells, × 10^9^/L	10.4 ± 3.0	9.9 ± 2.9	10.5 ± 3.1	0.557
Hemoglobin, g/L	132.2 ± 14.8	130.9 ± 19.1	132.7 ± 13.2	0.724
Creatinine, µmol/L	79.8 ± 38.8	92.6 ± 68.7	75.0 ± 18.0	0.947
D-dimer, mg/L	2.9 ± 2.3	2.8 ± 3.1	2.9 ± 2.3	0.929
TAD/TLD ratio	1.46 ± 0.16	1.47 ± 0.16	1.46 ± 0.16	0.843
Procedure success	44 (100.0)	12 (100.0)	32 (100.0)	NA
aTEVAR	22 (50.0)	6 (50.0)	16 (50.0)	1.000
Endograft number	1.2 ± 0.5	1.3 ± 0.6	1.2 ± 0.4	0.691
Stent material				
ePTFE	12 (27.3)	2 (16.7)	10 (31.3)	0.333
Polyester	36 (81.8)	11 (91.7)	25 (78.1)	0.300
Proximal landing zone 2	12 (27.3)	2 (16.7)	10 (31.3)	0.333
LSA coverage	0 (0.0)	0 (0.0)	0 (0.0)	NA
LSA reconstruction	12 (27.3)	2 (16.7)	10 (31.3)	0.333
LSA endograft implanted	2 (16.7)	1 (8.3)	1 (3.1)	0.460
Castor single-branch stent	1 (2.3)	0 (0.0)	1 (3.1)	1.000
External fenestration	1 (2.3)	0 (0.0)	1 (3.1)	1.000
LSA-LCA bypass	8 (18.2)	1 (8.3)	7 (21.9)	0.300

DM, Diabetes mellitus; nDM, Non-diabetes mellitus; SBP, Systolic blood pressure; DBP, Diastolic blood pressure; COPD, Chronic obstructive pulmonary disease; TAD, Total aortic diameter; TLD, True lumen diameter; aTEVAR, Acute thoracic endovascular aortic repair; LSA, Left subclavian artery; LCA, Left carotid artery.

### TEVAR details

TEVAR procedure was successfully performed in all patients (Table 1). The number and material of the endografts were similar between the two groups. In the case of proximal landing zone 2, we performed no coverage of left subclavian artery (LSA). The incidence of LSA reconstruction during the operation was higher in the nDM group, although there was no statistically significant difference between the two groups (DM group: 16.7%; nDM group: 31.3%, *p* = 0.333). Chimney technique, external fenestration, LSA-left carotid artery (LCA) bypass, or a Castor single-branched graft was employed to reconstruct LSA.

### Outcomes at hospital and 1-year follow-up

**Figure 2:**
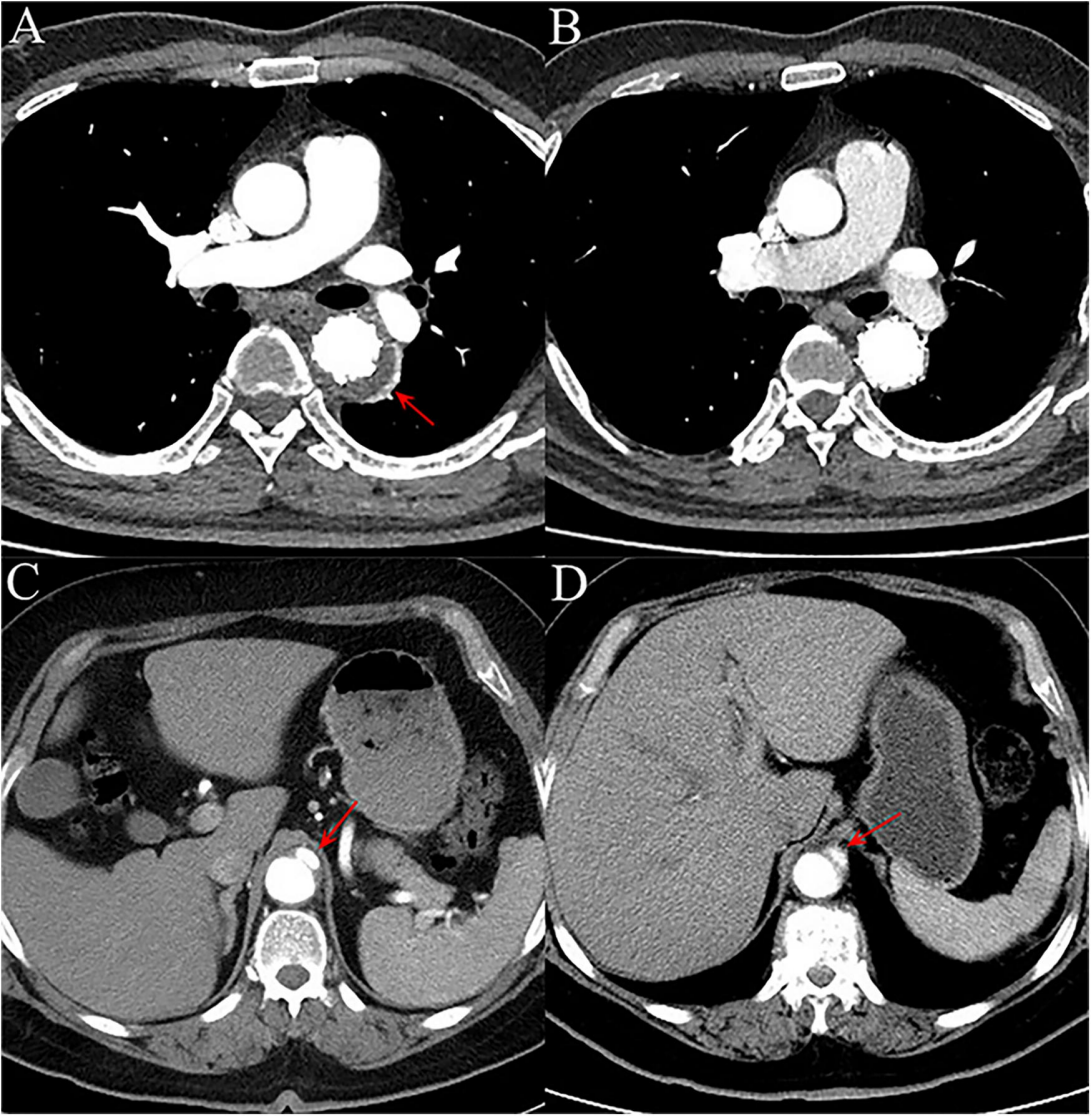
**(A)** on aortic computed tomography angiography (CTA) performed within 30 days after the procedure, a type Ib endoleak (red arrow) was observed in one patient, with contrast agent leaking into the aortic mid-layer from the distal landing zone of the endograft. **(B)** The endoleak had disappeared by the 1-year follow-up CTA. **(C)** In another patient, CTA within 30 days post-operation revealed a new-onset focal aortic dissection (red arrow) originating from the distal landing zone of the endograft, without involvement of major branch vessels. **(D)** At the 1-year follow-up, the aortic dissection (red arrow) remained stable in extent, with no significant progression.

Postoperative outcomes within 30-days are shown in Table 2. The measurements of postoperative TAD/TLD ratio before discharge were available for most patients, and there was no difference between the two groups (1.26 ± 0.16 for DM vs 1.25 ± 0.12 for nDM, *p* = 0.927). Overall, the incidence of postoperative MAE within 30-days was slightly higher in the nDM group, with no statistically significant difference was found (16.7% for DM vs 25.0% for nDM, *p* = 0.557). Only one death occurred in the nDM group, and there was no significant difference between the DM and nDM groups. Aortic rupture, one of the serious complications associated with the TEVAR procedure, was not observed in our study. On postoperative CTA before discharge, the incidence of endoleak type Ib and new-onset AD were also similar between the two groups, and no reintervention was needed (Figure 2). There were two cases of stroke and one case of SCI in the nDM group, while no cases of stroke or SCI were observed in the DM group. There were no significant inter-group differences, and all the three patients recovered before discharge. The rate of other postoperative complications, such as ACI, was also similar between the two groups.

**Table 2:** Postoperative outcomes within 30-days and during 1-year follow-up.

Variables	Overall	DM *n* = 12	nDM *n* = 32	*P-value*	Hazard ratio [95% CI]
CTA available before discharge	40 (90.9)	12 (100.0)	28 (87.5)	0.562	NA
TAD/TLD ratio	1.25 ± 0.13	1.26 ± 0.16	1.25 ± 0.12	0.927	NA
Hospitalization days	17.3 ± 6.7	18.5 ± 6.6	16.9 ± 6.7	0.486	NA
MAE before discharge	10 (22.7)	2 (16.7)	8 (25.0)	0.557	0.600 [0.108–3.338]
All-cause mortality	1 (2.3)	0 (0.0)	1 (3.1)	1.000	NA
Aortic rupture	0 (0.0)	0 (0.0)	0 (0.0)	NA	NA
Endoleak type Ib	2 (16.7)	1 (8.3)	1 (3.1)	0.460	2.818 [0.162–49.008]
New-onset aortic dissection	3 (6.8)	1 (8.3)	2 (6.3)	0.807	1.364 [0.112–16.577]
Reintervention	0 (0.0)	0 (0.0)	0 (0.0)	NA	NA
Stroke	2 (16.7)	0 (0.0)	2 (6.3)	1.000	NA
Spinal cord ischemia	1 (2.3)	0 (0.0)	1 (3.1)	1.000	NA
Acute cardiac insufficiency	1 (2.3)	0 (0.0)	1 (3.1)	1.000	NA
Follow-up available	43 (97.7)	12 (100.0)	31 (96.9)	1.000	NA
CTA available	28 (63.6)	7 (58.3)	21 (65.6)	0.654	0.733 [0.188–2.857]
MAE during follow-up	3 (6.8)	1 (8.3)	2 (6.3)	0.807	1.364 [0.112–16.577]
Endoleak type Ib	2 (16.7)	1 (8.3)	1 (3.1)	0.460	2.818 [0.162–49.008]
New-onset aortic dissection	1 (2.3)	0 (0.0)	1 (3.1)	1.000	NA
TAD/TLD ratio	1.15 ± 0.09	1.09 ± 0.04	1.17 ± 0.10	0.032	NA

DM, Diabetes mellitus; nDM, Non-diabetes mellitus; CI, confidence interval; CTA, Computed tomography angiography; TAD, Total aortic diameter; TLD, True lumen diameter; MAE, Major adverse events.

**Figure 3:**
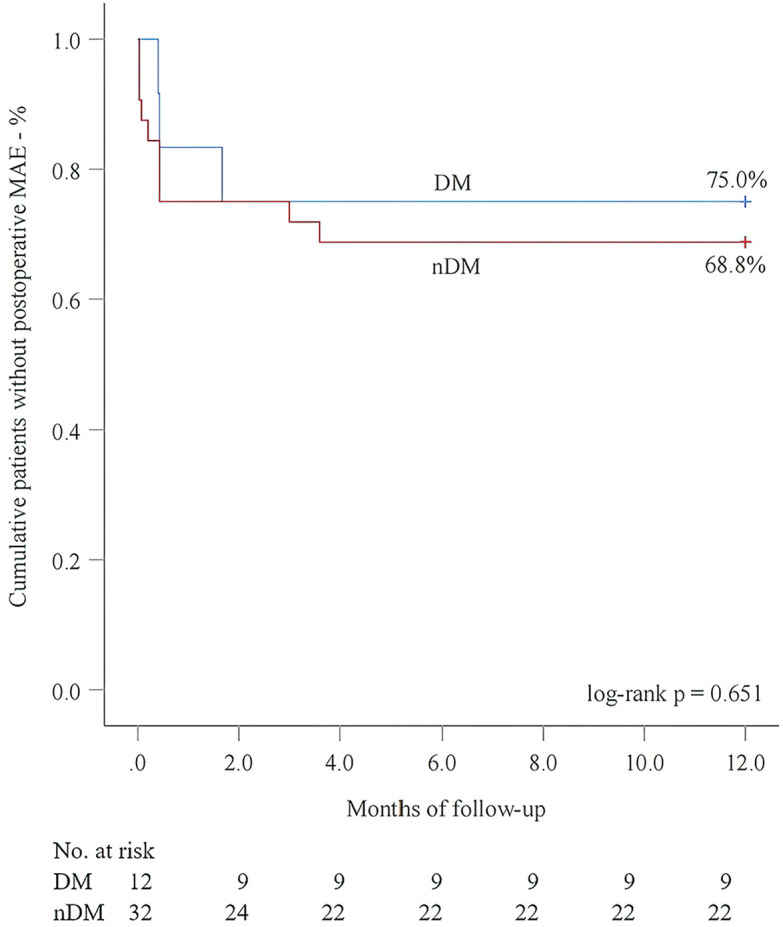
Kaplan–meier analysis for postoperative major adverse events (MAE) in relation to diabetes mellitus (DM).

**Figure 4:**
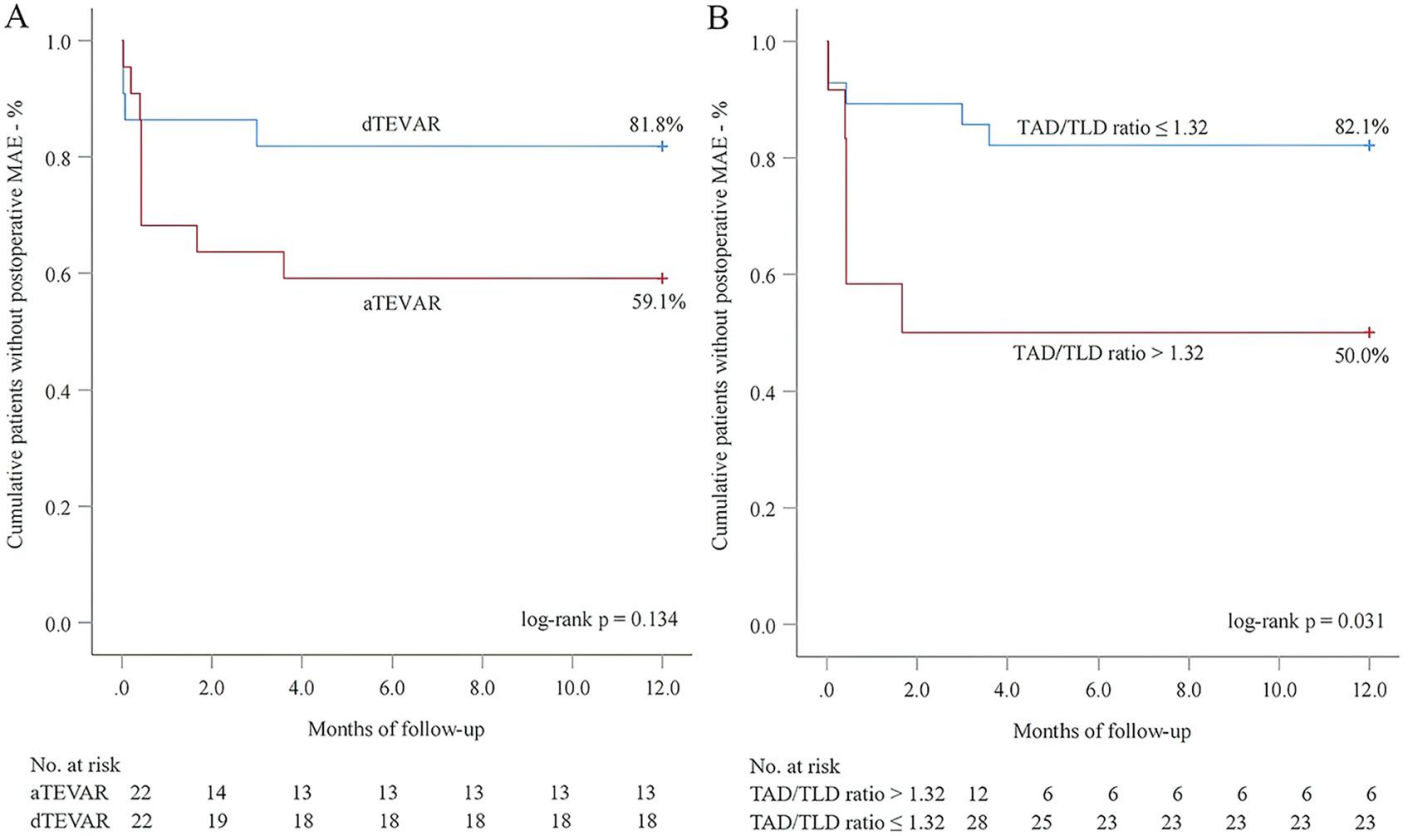
Kaplan–meier analysis for the association of major adverse events (MAE) with endovascular repair timing **(A)** and total aortic diameter (TAD)/true lumen diameter (TLD) ratio **(B).**

Next, we performed 1-year follow-up for most patients (DM group: 100.0%; nDM group: 96.9%, *p* = 1.000) (Table 2). Overall, a similar incidence of MAE was investigated between the DM and nDM groups during 1-year follow-up. Endoleak type Ib occurred in one case in each group, and one new-onset AD occurred in the nDM group. However, none of the three patients required reintervention. On aortic CTA, the TAD/TLD ratio was significantly lower in the DM group (DM group: 1.09 ± 0.04; nDM: 1.17 ± 0.10, *p* = 0.032). Due to varying levels of patient compliance, a number of patients did not return to the hospital for 1-year CTA examination. To assess whether this introduced potential selection bias, we conducted a comparison between patients who completed 1-year CTA follow-up and those who did not. No significant differences were observed between the two groups (Table 3). In the K-M analysis, the DM group had a slightly higher proportion of patients without postoperative MAE. However, the log-rank test showed no significant difference between the DM and nDM groups (75.0% for DM vs 68.8% for nDM, *p* = 0.651) (Figure 3). In previous studies, aTEVAR has been associated with higher rates of postoperative MAE (7, 8). We also found that aTEVAR increased the incidence of MAE in our study; however, this increase was not statistically significant compared to dTEVAR (59.1% for aTEVAR vs 89.8% for dTEVAR, log-rank *p* = 0.134) (Figure 4A). Finally, we determined the optimal cutoff point of the TAD/TLD ratio as 1.32 in accordance with previous study (7), and a significantly lower proportion of patients without MAE was observed in the group with a TAD/TLD ratio >1.32 (50.0% vs 82.1%, *p* = 0.031) (Figure 4B). Then, we performed a multivariate Cox regression analysis, and found that the TAD/TLD ratio >1.32 was independently associated with a higher incidence of postoperative MAE (HR, 3.798; 95% CI, 1.038–13.901; *p* = 0.044) (Table 4).

**Table 3:** Comparison of postoperative outcomes between patients with and without 1-year CTA follow-up.

Variables	Overall	With CTA *n* = 28	Without CTA *n* = 16	*P-value*	Hazard ratio [95% CI]
DM	12 (27.3)	7 (25.0)	5 (31.3)	0.732	0.733 [0.188–2.857]
nDM	32 (72.7)	21 (75.0)	11 (68.8)	0.732	1.364 [0.350–5.213]
MAE at 1-year follow-up	3 (6.8)	3 (10.7)	0 (0.0)	0.290	NA

CTA, Computed tomography angiography; CI, confidence interval; DM. Diabetes mellitus; nDM, Non-diabetes mellitus; MAE, Major adverse events.

**Table 4:** Multivariate analysis of risk factors for postoperative MAE.

Variables	Hazard ratio	95% CI	*P-value*
DM	0.777	0.190–3.178	0.725
aTEVAR	1.910	0.501–7.276	0.343
Proximal Landing zone 2	2.062	0.528–8.050	0.298
TAD/TLD ratio > 1.32	3.798	1.038–13.901	0.044

MAE, Major adverse events; CI, confidence interval; DM. Diabetes mellitus; TAD, Total aortic diameter; TLD. True lumen diameter.

## Discussion

In this study, the proportion of patients with DM was 27.3%, which is similar to the values reported in previous studies (9–12). As one of the most important prognostic indicators, all-cause mortality has been reported to be higher in patients with DM after TEVAR (12, 24, 25), while a similar result was also observed in some other studies (11, 25, 26). Another study reported that dietary therapy for DM was associated with significantly higher mortality during hospitalization and follow-up (11). In our study, 10 patients received oral medications alone, while two patients received insulin therapy in combination with oral medications in the DM group. During the 30-day postoperative period, one postoperative death was observed in patients without DM, while no deaths occurred in patients with DM. There was no significant inter-group difference. Such a result might be attributed to the absence of dietary management in the DM group.

In addition to mortality, SCI and stroke are considered serious complications following the TEVAR procedure. In our study, there were two cases of stroke and one case of SCI in the nDM group, while no such cases were observed in patients with DM. Although no significantly statistical inter-group differences were observed and the three patients recovered before discharge, DM appeared to decrease the incidence of nervous system complications following the TEVAR procedure. In some previous studies, DM acted as an independent risk factor of SCI and stroke after TEVAR (25, 27). However, the incidence of postoperative stroke was also recognized to be associated with extensive manipulation in the aortic arch region, which might occur during procedures such as LSA revascularization or coverage of LSA (27). In our study, no LCSA coverage was performed during operation. Moreover, there was a lower rate of LSA reconstruction in the DM group, decreased operations at the aortic arch, and resulted in the relatively lower incidence of nervous system complications.

Overall, compared with patients with DM, a higher incidence of postoperative MAE was investigated in patients without DM, but it did not reach statistical significance. This result has also been reported in some studies (10, 11). Traditionally, the aortic remodeling degree was considered to be positively associated with the prognostic outcomes of TEVAR procedure (7, 13, 15), which was confirmed by our study. We determined poor aortic remodeling degree as a TAD/TLD ratio >1.32 after intervention. Through multivariate regression analysis, we found that this ratio was significantly associated with a higher incidence of postoperative MAE. aTEVAR is negatively associated with the aortic remodeling degree, and it increases the rate of postoperative MAE (7, 8). Nevertheless, in our study, comparable patients underwent aTEVAR between both groups, and aTEVAR did not increase the rate of MAE in the K-M analysis.

Although postoperative TAD/TLD ratio was comparable between patients with and without DM within 30-days after TEVAR, a significantly lower data was investigated in the DM group at 1-year follow-up. In other words, patients with DM might achieve a better long-term aortic remodeling, resulting in a relatively lower incidence of postoperative MAE. This may be explained by the protective effects of DM on the aortic wall. Hyperglycemia promotes the formation of extracellular matrix and reduces its degradation in the aorta. Additionally, patients with DM typically have a greater intima-media thickness in the aorta, which helps to mitigate aortic wall stress (12, 28, 29). The putative protective effect of hyperglycemia on the aortic wall may also prohibit the development and progression of aortic aneurysm and aortic dissection, subsequently decreasing some serious complications such as mortality and aortic rupture (10, 16–18).

First, the relatively small sample size, particularly within subgroup analyses, may have limited the statistical power to detect significant differences in certain outcome measures. Second, this was a retrospective study conducted at two tertiary centers, which may introduce selection bias and limit the generalizability of the findings. Additionally, mid- to long-term follow-up data were not available for all patients, which may affect the assessment of durability outcomes. Future prospective, multicenter studies with larger cohorts and longer follow-up periods are warranted to validate these findings.

## Conclusion

In patients with acute type B IMH undergoing the TEVAR procedure, DM slightly decreases the incidence of postoperative MAE. This effect may be attributed to the significantly higher degree of aortic remodeling observed after intervention. Moreover, a TAD/TLD ratio >1.32 independently predicts the incidence of postoperative MAE.

## Data Availability

The original contributions presented in the study are included in the article/Supplementary Material, further inquiries can be directed to the corresponding author.
